# Evaluation of Novel Rapid Analytical Methods to Categorize Extra Virgin Olive Oil Based on the Coulometrically Determined Antioxidant Capacity and on the Spectrophotometric Assessment of Phenolic Compounds

**DOI:** 10.3390/molecules28073108

**Published:** 2023-03-30

**Authors:** Francesco Siano, Gianluca Picariello, Anna Sofia Sammarco, Giuseppe Celano, Tonino Caruso, Ermanno Vasca

**Affiliations:** 1 Istituto di Scienze dell’Alimentazione, Consiglio Nazionale delle Ricerche, Via Roma 64, 83100 Avellino, AV, Italy; francesco.siano@isa.cnr.it; 2 Dipartimento di Chimica e Biologia “A. Zambelli”, Università degli Studi di Salerno, Via Giovanni Paolo II 132, 84084 Fisciano, SA, Italy; ansammarco@unisa.it (A.S.S.); tcaruso@unisa.it (T.C.); evasca@unisa.it (E.V.); 3 Dipartimento di Farmacia, Università degli Studi di Salerno, Via Giovanni Paolo II 132, 84084 Fisciano, SA, Italy; gcelano@unisa.it

**Keywords:** extra virgin olive oil, phenolic compounds, coulometrically determined antioxidant capacity, fast blue BB assay, EFSA health claim, antioxidants

## Abstract

The lack of a practical “fit for the purpose” analytical protocol is the main limitation that has hampered the exploitation of the EFSA analytical health claim on the extra virgin olive oil (EVOO) biophenols, more than ten years since its introduction. In this work, two analytical methods recently developed in our laboratories for categorizing EVOO have been evaluated on a set of 16 samples from Cilento (Campania Region, southern Italy) and compared to other commonly used quality indexes. The Coulometrically Determined Antioxidant Capacity (CDAC) is associated with the component responsible for the health-promoting properties and oxidative stability of EVOO. The Fast Blue BB (FBBB) assay consists of the spectrophotometric (420 nm) determination of biophenols-FBBB diazonium coupling products generated in unfractionated EVOO. The FBBB assay and HPLC-UV reference method provide values highly correlated to each other. Fourteen of sixteen EVOO samples with CDAC > 10 mmol kg^−1^ and FBBB absorbance > 0.5 had HPLC-determined biophenols > 250 mg kg^−1^, and therefore eligible for the EFSA health claim. Consistently, two EVOO samples with HPLC-determined biophenols < 250 mg kg^−1^ had CDAC values and FBBB absorbance below the respective thresholds. CDAC and FBBB assays are proposed individually or in combination as methods to categorize EVOO samples in alternative to HPLC-UV.

## 1. Introduction

Phenolic compounds of extra virgin olive oil (EVOO), also referred to as biophenols, include a heterogeneous class of compounds mainly represented by tyrosol (*p*-hydroxyphenyl-ethanol), hydroxytyrosol (3,4-di-hydroxyphenyl-ethanol), secoiridoids such as oleuropein, ligstroside and its derivatives, phenolic acids, flavonols, and lignans [1]. Biophenols are minor components of EVOO as they are part of the 1–2% non-glyceride compounds, and alongside other minor components such as tocopherols and carotenoids they contribute to many of the health-promoting properties more or less consistently attributed to EVOO [2]. The qualitative and quantitative composition of biophenols varies depending on olive genotype and ripeness, agroclimatic conditions, oil extraction process, and storage conditions [3,4]. The level of biophenols, which can cover a relatively wide range between 50 and 800 mg kg^−1^, affects the antioxidant properties and chemical stability of EVOO [5] as well as the sensory traits because these compounds determine the bitterness, pungency, and astringency of high-quality EVOO [6,7]. The European Commission Regulation n. 2568/1991 [8], amended and revised by the EU Regulation 1604/2019 [9] laying down the quality parameters of EVOO and relevant methods of analysis, does not include the content of biophenols as a possible index to categorize EVOO. Transposing the recommendation of the European Food Safety Agency (EFSA) [10], the EU Regulation 432/2012 [11] has approved the use of a specific health claim for EVOO biophenols “Olive oil polyphenols contribute to the protection of blood lipids from oxidative stress”, which can be used to label EVOO containing at least 5 mg of hydroxytyrosol and its derivatives (e.g., oleuropein derivatives, ligstroside, and tyrosol) per 20 g of oil (polyphenols ≥ 250 mg kg^−1^). The health claim based on the content of biophenols could be a valuable tool to sub-segment high-quality oils within the EVOO category, thereby increasing nutritional benefits for consumers and promoting the interest for high quality productions [12]. Nevertheless, after more than 10 years since its introduction, the health claim is very seldom used on the label of commercial EVOO. Failure to appropriately address the health claim has detrimental economic consequences for high quality EVOO producers, competitiveness of food marketers, and consumers’ awareness of quality, as recently assessed [13]. The unavailability of a rapid and easy method to quantify phenolic compounds and to assess conformity during EVOO shelf-life is likely the main drawback limiting the commercial exploitation of the health claim [14,15]. For accurate quantification of EVOO biophenols the International Olive Council (IOC) recommends the HPLC-UV analysis of hydroalcoholic extracts (80% aqueous methanol, *v*/*v*), using syringic acid as the internal standard [16], which is a technically laborious, cost- and time-consuming method and it is not free from limitations [17]. The method has been revised in 2017 [18], and even more recently, in 2022, two different methods have been endorsed [19]. These methods differ in the liquid–liquid or solid phase extraction procedures, whereas only minor adjustments have been introduced for the HPLC-UV or HPLC-DAD phase of the analysis, mainly aimed at improving accuracy and specificity. However, the practical applicability of the assay has not increased significantly. To restrain the assay only to those biophenols designated by the EFSA health claim and to shorten the time of analysis, two groups have proposed the HPLC determination of hydroxytyrosol and tyrosol alone following their release by acidic hydrolysis [17,20]. This method is accurate and time-effective, but it is unlikely to make routine the determination of biophenols in oil because it does not overcome many of the drawbacks of the HPLC analysis.

Recently, our group developed two independent rapid techniques for categorizing EVOO, one based on the coulometrically determined antioxidant capacity (CDAC) [21] and the other on the spectrophotometric quantification of phenolic compounds after coupling with the Fast Blue BB diazonium salt in unfractionated oil [22].

Biophenols are key antioxidant compounds, although CDAC of EVOO includes the contribution of various additional components. In general, CDAC of EVOO with biophenols ≥ 250 mg kg^−1^ (15 ± 4 mmol e^−^ kg^−1^) is significantly higher than EVOO with biophenols < 250 mg kg^−1^ (10 ± 2 mmol e^−^ kg^−1^) [21]. However, EVOO samples with CDAC values comprised between 10 and 15 mmol e^−^ kg^−1^ should be classified with different methods, for instance by HPLC-UV. On the other hand, the Fast Blue BB (FBBB) assay provides values satisfactorily correlated to the HPLC determination of biophenols and could be a cost- and time-effective alternative to the method recommended by IOC [22]. The determination of CDAC and the FBBB assay do not require sophisticated or expensive instrumentation and chemicals, can be performed even by not particularly skilled personnel, and are suited for automatization and multiplexing. Notably, the analysis times are very short and are compatible with the need to classify EVOO soon after extraction, at the bottling stage.

In this work, the novel methods proposed were evaluated on EVOO samples collected in a small geographical area of southern Italy (Cilento, Province of Salerno, Campania Region). In particular, 14 EVOO samples with CDAC values covering the uncertainty range (i.e., 10–15 mmol e^−^ kg^−1^) and 2 with CDAD value < 10 mmol e^−^ kg^−1^ were selected for assaying multiple quality parameters. Biophenols in these samples were determined comparatively by HPLC-UV, FBBB, and Folin–Ciocalteau assays.

## 2. Results and Discussion

Following a pre-screening based on the measurement of CDAC of more than 50 EVOO samples, 14 EVOO samples with CDAC values covering the uncertainty range (i.e., 10–15 mmol e^−^ kg^−1^) were selected. Two additional EVOO samples (n. 15 and 16) with PCs-HPLC lower than 250 mg kg^−^^1^ were considered for comparison. Free acidity, PV, C_18:1_/C_18:2_ ratio, total biophenols determined with the FC method (PCs-FC) or with HPLC-UV (PCs-HPLC), DPPH inhibition, CDAC and FBBB values for the 16 EVOO samples considered in this study are listed in Table 1. The EVOO samples were not evaluated by a trained sensory panel. However, based on free acidity and PV, all the samples considered in this study can be classified as EVOO. Nevertheless, other parameters determined for these oils related to their quality cover relatively broad ranges, confirming that the EVOO category includes oils with an ample variety of traits and that conventional chemical analyses could be inadequate to sub-segment commercial products classified as EVOO.

### 2.1. HPLC-UV Analysis

**Figure 1:**
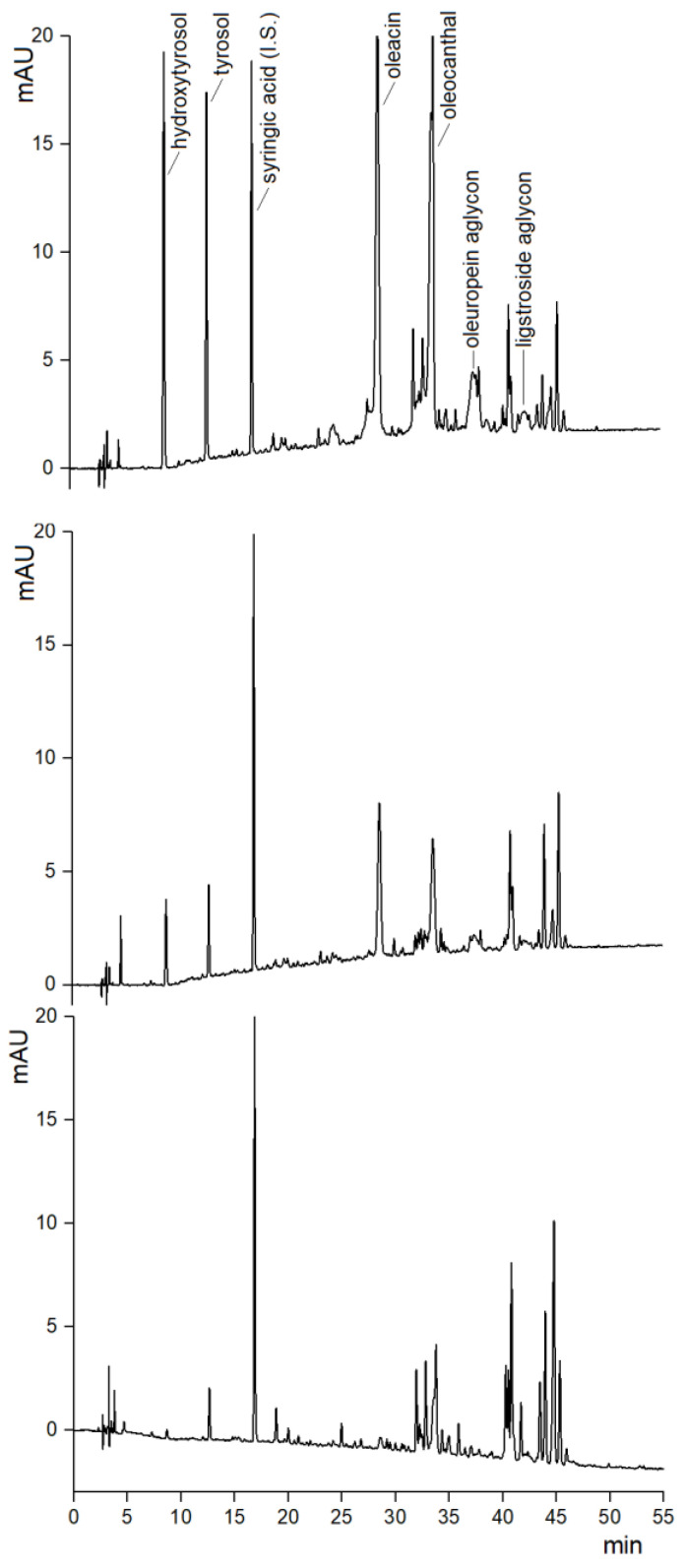
Example comparison among HPLC-UV (280 nm) chromatograms of three EVOO polar extracts: sample no. 3 (**up**), no. 5 (**middle**), no. 15 (**low**). The qualitative pattern of biophenols appeared rather conserved for samples no. 3 and no. 5, while no. 15 differed significantly. The quantitative balance of biophenols differed, while the peak area of syringic acid (internal standard) was unmodified (variability < 2%).

EVOO biophenols vary significantly depending on a multitude of genetic, pedoclimatic, and technological factors [3,4]. Moreover, they can vary during shelf-life depending on the storage conditions [6]. Among the samples analyzed, the biophenol patterns varied both on a qualitative and quantitative basis, although they came from a small geographic area and their antioxidant capacity, evaluated as CDAC, covered a relatively narrow range. As an example, HPLC-UV chromatograms (280 nm) of polar extracts from three EVOO samples, namely sample n. 3, 5 and 15, are shown in Figure 1. Comparing the chromatograms of samples n. 3 (PCs-HPLC 0.59 g kg^−^^1^) and 5 (PCs-HPLC 0.32 g kg^−^^1^), the different intensity of individual biophenols was evident, while the qualitative elution profiles remained substantially conserved. In contrast, the elution profile of sample n. 15 (PCs-HPLC 0.20 g kg^−^^1^) was clearly different, especially because the hydroxytyrosol and its derivatives (i.e., oleacin and oleuropein aglycone) were almost missing. The area of the internal standard (syringic acid) was practically unmodified, with variability < 2%, calculated as % relative standard deviation of the peak areas over the entire set of 16 EVOO samples.

### 2.2. Principal Component Analysis (PCA)

**Figure 2:**
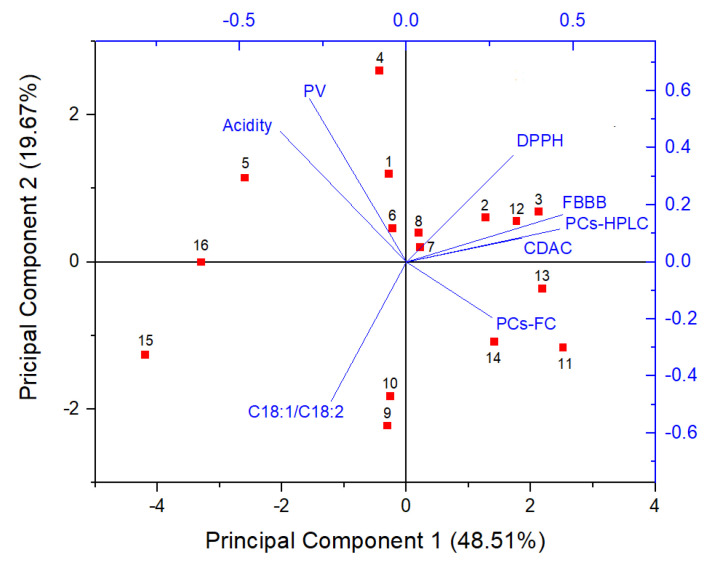
Principal Component Analysis (PCA) biplot for EVOO samples according to the eight sets of values evaluated in this study (acidity, PV, C_18:1_/C_18:2_, PCs-HPLC, PCs-FC, FBBB, CDAC, and DPPH). Samples are numbered as in Table 1.

A PCA analysis was performed to assess possible correlations among the different parameters determined in this study. The PCA biplot for EVOO samples according to the eight sets of values (acidity, PV, C_18:1_/C_18:2_, PCs-HPLC, PCs-FC, FBBB, CDAC, and DPPH) is shown in Figure 2. The dimensionality was reduced to two principal components, which explained 68.18% of the total variability (PC1 = 48.51% and PC2 = 19.67%). Scores were heavily distributed over the plot, exhibiting no substantial clustering.

The angles between loading vectors reflected a lack of interdependence between variables, except for a significant association between FBBB and PCs-HPLC, which will be discussed later. As expected, free acidity and PV, which are two indexes used for classifying olive oil, were independent of the parameters related to the antioxidant capacity or the content of biophenols. The oleic (C_18:1_)/linoleic (C_18:2_) acids ratio is an additional index related to the nutritional quality and oxidative stability of olive oils [23]. It is controlled by genotypic factors through oleate desaturase isoforms [24] but it can be importantly affected by pedoclimatic conditions [25]. In spite of the homogeneous origin of the EVOO samples analyzed in this study, the oleic/linoleic acids ratio varied within an ample range (4.3–14.0). However, as evident from PCA analysis, this parameter was practically unrelated to the others. On the contrary, CDAC values seemed correlated to both FBBB and PCs-HPLC. This was likely an apparent effect resulting from the reduced dimensionality in the PCA analysis, because in pairwise association CDAC was substantially uncorrelated to either FBBB (R^2^ = 0.460) or PCs-HPLC (R^2^ = 0.538). The CDAC values determined in this study are the means of three independent measurements, renewing the KBr solution each time. In this way, the inter-measurement variability was very low (<5%), while CDAC values tended to be slightly lower than those obtained in our previous work [21]. Overall, 14 out 14 samples with CDAC > 10 mmol e^−^ kg^−1^ exhibited PCs-HPLC > 250 mg kg^−1^ and therefore eligible for the health claim, while the two samples with CDAC < 10 mmol e^−^ kg^−1^ had PCs-HPLC < 250 mg kg^−1^. These results strengthen our previous indications that 10 mmol e^−^ kg^−1^ could be a threshold CDAC value for distinguishing EVOO samples eligible from not eligible for the health claim [21]. Clearly, it remains necessary to establish a certain tolerance range because CDAC could vary depending on several compounds other than biophenols (e.g., tocopherols). Nonetheless, CDAC should be considered a candidate method for classifying EVOO samples based on the antioxidant capacity rather than on the content of biophenols alone. Such a quality index could be as valuable as the content of biophenols, as it incorporates either the health-promoting effects or the oxidative stability of EVOO, which are both associated with the antioxidant capacity of the EVOO polar fraction [26]. Other authors have found a significant correlation between EVOO phenolic compounds and antioxidant capacity determined with ferric reducing antioxidant power (FRAP) and Trolox equivalent antioxidant capacity (TEAC) assays. However, the correlation patterns were not stable as they appeared to be dependent on the qualitative profile of biophenols [27]. Apart from the executive simplicity and the time-cost effectiveness of the test, the greatest advantage of CDAC compared to other methods for the determination of antioxidant capacity resides in its sensitivity as well as in the relative selectivity that renders it unaffected by interfering species.

Although the Folin–Ciocalteu method is still widely used for determining biophenols in EVOO especially for research purposes, the PCs-FC values were uncorrelated with PCs-HPLC, DPPH, and CDAC, contrasting with previous data that highlighted significant correlation among several radical scavenging tests and between individual assays and PCs-FC [28]. Herein findings suggest that the Folin–Ciocalteu assay is inadequate for an accurate determination of biophenols in EVOO and yields results discrepant with those obtained with the IOC HPLC method, likely because it is biased by unpredictable amounts of interfering compounds [29] and by a variable response of individual phenolic compounds [30,31]. The lack of mutual correlation between the Folin–Ciocalteu and DPPH assays and their incongruity with the HPLC-based determinations confirm the unsuitability of these methods for assessing conformity of EVOO samples to the health claim requirements.

### 2.3. FBBB—PCs-HPLC Correlation and FBBB—CDAC Association

**Figure 3:**
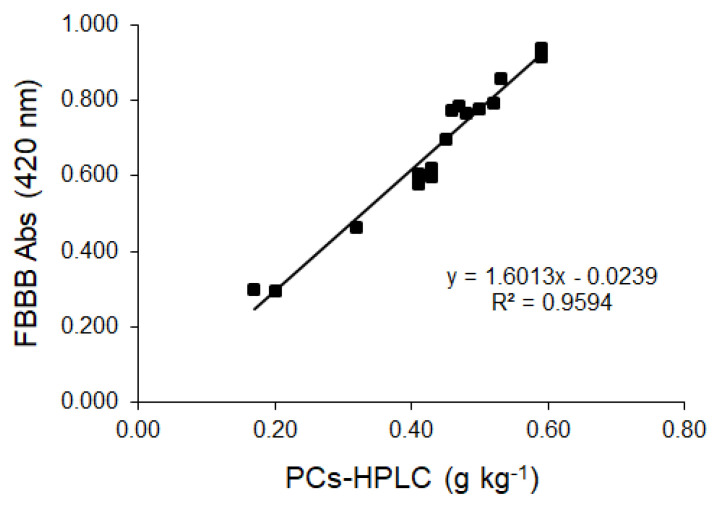
Pairwise correlation between Fast Blue BB (FBBB) absorbance and total biophenols determined with the HPLC-UV method (PCs-HPLC) for the 16 EVOO samples analyzed. Values are means of three replicates. Relative standard deviation (% RDS) was <5% in all cases and error bars have been omitted.

The pairwise correlation between FBBB absorbance and PCs-HPLC had R^2^ = 0.959 (Figure 3), confirming an evident association between the two parameters.

If PCs-HPLC were expressed as mg kg^−^^1^ oil, the slope of the correlation line was 0.0016 which is in close agreement with the one obtained in our previous article (0.0019) [22]. The FBBB assay is specific for biophenols as it is affected at a very low extent by interfering compounds other than biophenols, tocopherols included. Just like the IOC-recommended HPLC-UV method, the FBBB assay incorporates in the evaluation also a small percentage of phenolic compounds other than hydroxytyrosol and tyrosol, both as free and esterified forms. However, the discrepancy of these methods in comparison to the HPLC-based determination of hydroxytyrosol and tyrosol following acidic hydrolysis should not exceed 5% [17].

**Figure 4:**
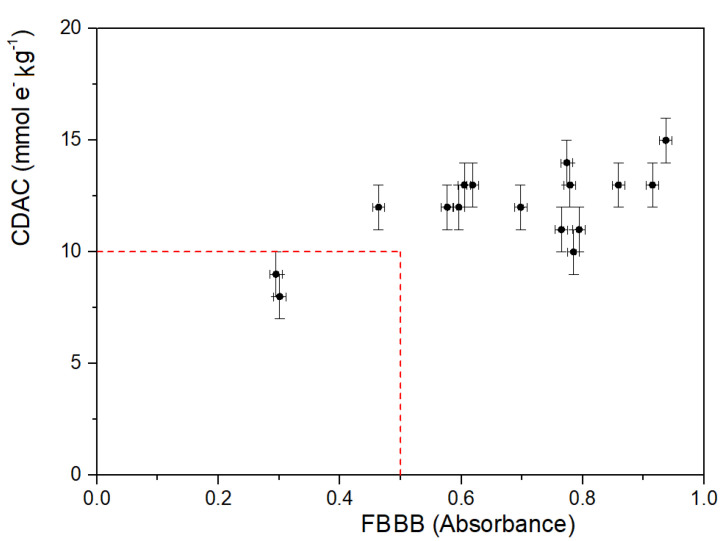
Plot of coulometrically determined antioxidant capacity (CDAC) values vs. Fast Blue BB (FBBB) absorbance. Values are means of three replicates. Error bars represent the estimated precision as standard deviation.

A plot associating CDAC and FBBB assay is shown in Figure 4. In spite of a substantial lack of correlation between the parameters, the plot summarizes the concept that CDAD was higher than the threshold values of 10 mmol e^−^ kg^−^^1^ and simultaneously FBBB absorbance (420 nm) was higher than 0.5 for all the investigated EVOO samples with PCs-HPLC > 250 mg kg^−^^1^. The CADC and FBBB values below these thresholds for the two EVOO samples with PCs-HPLC 0.20 and 0.17 g kg^−^^1^ were consistent with this classification. Therefore, CDAC and FBBB assays could be used individually or in combination in alternative to HPLC-UV as rapid and cheap methods to categorize EVOO samples. In particular, CDAC could be useful for a quick pre-screening, but its routine commercial application would require a modified formulation the health claim. On the contrary, the FBBB assay can determine reliably the concentration of biophenols in alternative to the HPLC-UV analysis. Interestingly, FBBB could be used as an indicative colorimetric method for assessing the conformity to the health claim requirements, limiting the use of a spectrophotometer only to a small set of EVOO samples with uncertain attribution [22].

## 3. Materials and Methods

EVOO samples were obtained from a local consortium (Nuovo Cilento S.c.a.r.l., San Mauro Cilento), which collects oil from several farms located in the Cilento area, Province of Salerno, Campania Region (Italy). In all cases, the olives were harvested early, at veraison, and cold pressed within 24 h since harvesting at the same mill with continuous extraction plant. EVOO was sampled soon after extraction by cold pressing at local oil mills during the campaign 2022 (October–November). HPLC-grade solvents and high purity chemicals were purchased from Merck-Sigma (Milan, Italy). More than 50 samples were quickly screened measuring the CDAC values. For this study, 14 EVOO samples with CDAC values covering the uncertainty range (10–15 mmol e^−^ kg^−^^1^) and 2 EVOO samples with CDAC value < 10 mmol e^−^ kg^−^^1^ were selected for further analyses.

### 3.1. Acidity and Peroxide Value Analysis

Total acidity and peroxide value were determined by conventional titrimetric methods [32]. The acidity degree, expressed as mass percentage (%, *w*/*w*) of free oleic acid, was determined with a classical acid-base volumetric titration in diethyl ether:ethanol (1:1), using phenolphthalein as the indicator. Peroxide value (PV), expressed as milli-equivalents O_2_ (mEqO_2_) kg^−1^ of oil, was determined by titrating iodine liberated from potassium iodide with standard 0.02 M Na_2_S_2_O_3_, using a 1% starch solution as the indicator.

### 3.2. Extraction of Polar Compounds from EVOO Samples

Polar compounds were extracted from 2.0 g of EVOO samples with 6 mL of 80% (*v*/*v*) aqueous methanol in ultrasonic bath for 30 min at room temperature. The two-phase system was then centrifugated for 20 min at 5000× *g* and the polar solution (upper layer) was filtered on disposable 0.22 µm nylon (Merck-Millipore, Darmstadt, Germany). The filtrate was stored in glass vials at −26 °C until further analysis. For RP-HPLC analysis of biophenols the extraction solution was previously spiked with syringic acid as the internal standard.

### 3.3. DPPH Radical Scavenging Capacity

The radical-scavenging capacity was determined by spectrophotometric assay after combining 100 µL of polar extracts to 2.4 mL of freshly prepared 0.1 mM DPPH radical in ethanol and incubating the mixture for 30 min at room temperature in the dark. Absorbance was determined at 517 nm using a GE-Healthcare Ultrospec 2100 UV-Vis (Uppsala, Sweden) spectrophotometer against pure ethanol. DPPH radical-scavenging capacity was calculated as the % inhibition (% I) with the following formula:

% I = [(A_DPPH_ − A_s_)/A_DPPH_] × 100

where A_DPPH_ is the absorbance of the DPPH radical solution and A_s_ is the absorbance after radical inhibition with polar extracts from EVOO samples [33].

### 3.4. Determination of Total Phenolic Compounds with the Folin–Ciocalteu Method (PCs-FC)

Total phenolics were determined with the modified Folin–Ciocalteu (FC) method [34]. Briefly, in a 3 mL plastic cuvette were added consecutively: 2300 µL of distilled water, 50 µL of FC reagent diluted 1:2 with water, 50 µL of oil extract and after 3 min, 100 µL of a saturated Na_2_CO_3_ solution. After 90 min of incubation in the dark, the absorbance was measured at 765 nm (GE-Heathcare Ultrospec 2100 UV-Vis). Total phenolics were quantified against a calibration curve (R^2^ > 0.99) built with gallic acid (≥99% purity). Results were expressed as mg of gallic acid equivalent (GAE) per kg of oil (mg_GAE_ kg^−1^ oil).

### 3.5. CDAC

CDAC values were determined as previously detailed [21]. Briefly, the determinations were performed in a 150 mL flat bottom, closed glass vessel with four 14/23 normalized ground glass necks, three of which served to place the electrodes and the latter for sample introduction. For each CDAC measurement, 500 µL of EVOO extracts was used. After each determination, Pt electrodes are washed with 1/1 (*v*/*v*) 65% HNO_3_/H_2_O and repeatedly rinsed with double distilled water. In our previous work, the CDAC values were the median of five consecutive measurements without replacing the KBr solution for the electro-generation of bromine. To increase repeatability and accuracy, in this study the CDAC values are the mean of three independent determinations, which were performed by renewing the KBr solution each time.

### 3.6. Fast Blue BB

Fast Blue BB (FBBB) determinations were performed on unfractionated EVOO samples as previously detailed [22]. To this purpose, 2 mL of freshly prepared FBBB diazonium salt (0.1% *w*/*v* in ethanol) and 2 mL of 5% (1.25 M) NaOH were sequentially added to 1.0 g of EVOO samples in disposable 15 mL plastic tubes. After vertexing for 2 min and incubation in ultrasonic bath for 20 min, the mixture was centrifuged for 10 min at 3500× *g*. The clear hydroalcoholic phase (lower layer) was collected with a Pasteur pipette and the absorbance was measured at the wavelength of 420 nm in disposable semi-micro 1.5 plastic cuvettes (Kartell Labware, Noviglio, Milan, Italy). Reaction schemes and formation of the chromophore are shown in [22].

### 3.7. GC Analysis of Fatty Acids

Fatty acid as methyl ester (FAME) were determined according to Siano et al. [35]. EVOO samples (0.200 g) were transferred into Pyrex test tubes with screw caps containing 2 mL of 1.25 N HCl−methanol solution and incubated in a water bath at 90 °C for 60 min. FAMEs were extracted with *n*-hexane, after the addition of 2 mL of distilled water. The organic phase was filtered using Millex 0.45 µm PVDF disposable syringe filters (EMD Millipore Corp., Billerica, MA, USA), and 1 µL was directly injected into a gas chromatograph Agilent 7890 (Agilent, Palo Alto, CA, USA) equipped with a FID, using a Supelco SP-2560 100 m × 0.25 mm × 0.20 µm capillary column with biscyanopropyl siloxane stationary phase (Merck-Sigma). Samples were introduced through a split–splitless injection system of an Agilent 7683B Series autosampler in split mode (ratio 1:100) at 260 °C. The oven temperature program started at 140 °C (held for 5 min) and linearly increased to 260 °C (4 °C min^−1^) up to the end of the analysis. FID temperature was 260 °C. Fatty acid composition of EVOO was obtained by comparison with the retention times of the standard mixture FAME 37 components (Merck-Sigma) and was expressed as a percentage area. Data were recorded and processed using Chemstation vers. B04.03 suite (Agilent).

### 3.8. HPLC-UV Determination of Total Phenolic Compounds (PCs-HPLC)

HPLC-UV of phenolic compounds PCs were determined by reversed phase-high performance liquid chromatography (RP-HPLC), adapting the International Olive Council (IOC) method for the determination of the olive oil biophenols [16]. To this purpose, the polar oil extracts (CH_3_OH:H_2_O, 80:20) were concentrated in a Savant speed-vac and diluted up to 1 mL with 0.1% trifluoroacetic acid (TFA). One-tenth of the polar oil extract was separated by RP-HPLC using a modular UltiMate 3000 chromatographer (Thermo Scientific, Inc., San Jose, CA, USA) equipped with a diode array detector (DAD). The stationary phase was a C18 reversed-phase column 250 × 2.0 mm i.d., 4 µm particle diameter (Jupiter Phenomenex, Torrance, CA, USA), kept at a 37 °C using a thermostatic oven. Separations were carried out at a 0.2 mL min^−^^1^ constant flow rate, applying a 5–65% gradient of the organic modifier (solvent B: acetonitrile/TFA 0.1%) in 5–65 min, following 5 min of isocratic elution at 5% B. After 65 min the % B was increased up to 100%. Solvent A was 0.1% TFA in HPLC-grade water. Separations were monitored at λ = 280 nm wavelengths and peaks were integrated using the Chromeleon 7.2.10 Software (Thermo Scientific). The tyrosol-to-syringic acid (internal standard) response factor was determined in agreement with the IOC method [10] and total phenolics were expressed as tyrosol equivalents.

### 3.9. Statistical Analysis

Measured values are averages of at least three replicate analyses. Relative standard deviation was lower than 5% in all the cases and it was omitted. Principal component analysis (PCA) and data plotting was performed with Origin v. 8.6 (OriginLab Co., Northampton, MA, USA).

## 4. Conclusions

Despite intensive and long-lasting research on the subject, the determination of biophenols in EVOO is still a challenge. The lack of a practical “fit for the purpose” method for establishing compliance with the requirements has strongly hampered the exploitation of the EFSA-approved health claim on EVOO biophenols, so that many producers, researchers, and traders have recommended substantial revisions to the regulation [36]. CDAC measured by coulometric titration with biamperometric endpoint detection is a cheap, rapid, and accurate method to estimate the antioxidant capacity of EVOO. Although CDAC values are not strictly correlated to the content of biophenols, these latter compounds are key contributors to the antioxidant capacity. For these reasons, CDAC is particularly adequate for a rapid screening of EVOO samples and can also be reliably used for EVOO categorization based on the antioxidant capacity, which is in turn related to the health-promoting properties and oxidative stability of oil. From the current results, 10 mmol e^−^ kg^−1^ could be proposed as a threshold value for discriminating EVOO samples eligible (CDAC > 10 mmol e^−^ kg^−1^) from not eligible (CDAC < 10 mmol e^−^ kg^−1^) for the health claim. However, a tolerance range should be carefully defined if CDAC is to be intended as a method substitutive of HPLC-UV for assessing compliance with the EFSA health claim. On the other hand, the FBBB assay selectively targets EVOO biophenols and is negligibly biased by other classes of compounds, thereby differing from many indirect methods that rely on the assessment of total phenolic compounds through their reducing, antioxidant, or radical scavenging properties. Data of this study support the use of FBBB assay on unfractionated oil as a rapid, cheap, and reliable method, alternative to HPLC-UV, to determine biophenols in EVOO and to assess eligibility for the EFSA health claim, as currently enacted.

## Figures and Tables

**Table 1:** Determination of free acidity (expressed as % oleic acid), peroxide values (PV), C_18:1_/C_18:2_ ratio, total phenolics by HPLC (PCs-HPLC) and Folin–Ciocalteu (PCs-FC) methods, Fast Blue BB (FBBB), coulometrically determined antioxidant capacity (CDAC) and DPPH radical scavenging activity (% I) for the EVOO samples evaluated in this study. Measured values are average of at least three replicate analyses. In all cases relative standard deviation was lower than 5%.

N.	Acidity (%)	PV (mEqO_2_ kg^−1^)	C_18:1_/C_18:2_	PCs-HPLC (g kg^−1^)	PCs-FC (g kg^−1^)	FB BB (Abs)	CDAC (mmol e^−^ kg^−1^)	DPPH (% I)
1	0.49	11	7.64	0.45	0.23	0.698	12	17
2	0.40	10	9.26	0.53	0.43	0.859	13	18
3	0.38	11	8.33	0.59	0.70	0.915	13	18
4	0.61	16	9.07	0.50	0.26	0.779	13	18
5	0.64	13	8.61	0.32	0.13	0.464	12	14
6	0.39	11	7.08	0.48	0.31	0.765	11	14
7	0.29	10	7.93	0.41	0.32	0.605	13	17
8	0.47	8	7.36	0.43	0.33	0.619	13	17
9	0.23	7	13.49	0.43	0.36	0.596	12	14
10	0.22	8	13.96	0.41	0.36	0.577	12	16
11	0.20	6	8.52	0.59	0.48	0.937	15	14
12	0.18	8	4.27	0.47	0.46	0.785	10	20
13	0.20	8	8.40	0.46	0.61	0.774	14	19
14	0.22	6	8.20	0.52	0.48	0.794	11	16
15	0.56	12	13.99	0.20	0.31	0.295	9	12
16	0.62	12	8.68	0.17	0.56	0.301	8	15

## Data Availability

Raw data are available upon request to the corresponding author.

## Sample Availability

Samples of the compounds are not available from the authors.
